# NK Cell Regulation in Cervical Cancer and Strategies for Immunotherapy

**DOI:** 10.3390/cells10113104

**Published:** 2021-11-10

**Authors:** Adriana Gutiérrez-Hoya, Isabel Soto-Cruz

**Affiliations:** 1 Molecular Oncology Laboratory, Cell Differentiation and Cancer Research Unit, FES Zaragoza, National University of Mexico, Batalla 5 de Mayo S/n Col. Ejército de Oriente, Mexico City 09230, Mexico; adrianagh85@hotmail.com; 2 Cátedra CONACYT, CONACYT, Avenida Insurgentes Sur 1582, Col. Crédito Constructor Del. Benito Juárez, Mexico City 03940, Mexico

**Keywords:** cervical cancer, NK cells, HPV, immune system, immunotherapy

## Abstract

Cervical cancer is one of the most prevalent gynaecological malignancies worldwide and is related to human papillomavirus (HPV) infection, viral persistence, progression, and invasion. Therefore, the immune response is linked to HPV status. Natural killer (NK) cells play a central role against virus-infected cells and tumours through a delicate balance between activating and inhibitory receptors and secretion of cytokines and chemokines. These cells also play a crucial role in tumour immunosurveillance. For these reasons, there is growing interest in harnessing NK cells as an immunotherapy for cervical cancer. These studies are diverse and include many strategies such as transferring activated autologous or allogeneic NK cells, improving the activation and cytolytic activity of NK cells using cytokines or analogues and modifying chimeric antigen receptors to increase specificity and targeting NK cells. However, research regarding the application of NK cells in immunotherapy is limited. This article focuses on recent discoveries about using NK cells to prevent and treat cervical cancer and the possibility of cellular immunotherapy becoming one of the best strategies to exploit the immune system to fight tumours.

## 1. Introduction

Cervical cancer is the fourth most common cancer with the highest incidence and mortality in women worldwide (Globocan 2020). High-risk human papillomavirus (HPVs) (mainly with HPV16 and HPV18) is the most significant risk factor for developing cervical cancer; other risk factors include various sexually transmitted infections, smoking, increased number of deliveries, and prolonged use of oral contraceptives. However, high-risk HPVs are associated with the development of other cancers such as vaginal, vulvar, head and neck, anal, oropharyngeal, and penile carcinomas [1,2,3]. The presence of HPV in the sexually active population is common. Although the introduction of vaccines has decreased the incidence of the most common high-risk HPV serotypes (HPV16 and 18), the incidence and mortality of cervical cancer continue to be high. However, not all high-risk HPV-positive individuals will develop cancer. In cervical cancer, the HPV generally establishes infection in the basal epithelial layer. Most of these infections are transient because the immune system is activated and eliminates them in a few years. However, 10–20% of HPV infections persist latently, leading to disease progression [4,5,6,7,8].

Typically, the immune system eliminates foreign agents, such as HPV in this case. How does an HPV infection end? When HPV reaches the basal epithelial layer, physical barriers play an important role in preventing infection due to defensins, mucoproteins, and an acidic pH that prevent the virus from entering the keratinocytes. However, HPVs frequently evade these mechanisms and infect target cells. When HPV infects target cells, they can be eliminated by cells of the innate immune response such as natural killer (NK) cells, which induce an inflammatory immune response to achieve the recruitment of more NK cells, macrophages, dendritic cells (DC), cells of Langerhans (LC), and NK T cells (NKT) at the site of infection. These cells also participate in exacerbating inflammation and activating adaptive immune response cells, where CD4, TCD8, and LcB T cells participate. However, HPVs have developed a variety of strategies to escape the innate and adaptive immune response. Some of these strategies involve avoiding the inflammatory process during viral infection by secreting anti-inflammatory cytokines, decreasing the expression of molecules associated with damage, decreasing the expression of MHC Class I molecules, suppressing the interferon pathway, among other strategies [9,10,11,12,13].

A preventive measure for HPV infection is prophylactic vaccines such as bivalents such as Cervarix (HPV18 and 16), tetravalent such as Gardasil (HPV 6, 11, 16 and 18) and nonavalent vaccines such as Gardasil 9 (HPV 6, 11, 16, 18, 31, 33, 45, 52 and 58). National vaccination programs remain effective in preventing persistent HPV infection. However, its immune protection is limited to specific types of HPV, and it does not offer universal protection against HPV infection, nor is it effective as a treatment for existing HPV infection. Recently, clinical studies were carried out on possible therapeutic vaccines to reinforce adaptive immunity mediated by CD4 and CD8 T cells against HPV. However, one aspect of immunotherapy that is of great interest is the use of NK cells. These studies are diverse and include many strategies such as transferring activated autologous or allogeneic NK cells, improving the activation and cytolytic activity of NK cells using cytokines or analogues and modifying chimeric antigen receptors to increase specificity and targeting NK cells [8,14,15].

### Cervical Cancer

Cervical cancer, the fourth most common cancer, is a gynaecological cancer with the highest frequency, incidence, and mortality worldwide. According to Globocan, there were an estimated 604,127 new cases and 341,831 cervical cancer deaths worldwide in 2020. HPV has been implicated in more than 90% of cervical squamous cell cancers worldwide. Together with the European Organization for Research on Genital Infections and Neoplasms and the National Institutes of Health Consensus Conference on Cervical Cancer in 1996, the World Health Association recognised HPV as a major cause of cervical cancer. However, other factors have been shown to contribute to cervical cancer development, such as the early onset of sexual activity, a high number of sexual partners, infrequent use of condoms, multiple pregnancies, and infections by pathogens such as chlamydia and the presence of viral HIV infections. Cervical cancer is both preventable and curable if detected early and treated adequately. However, the lack of access to HPV vaccination programs, early detection programs, and proper sexual education means this cancer has a higher incidence in developing countries and is detected in the late stages. Late detection of cervical cancer is a problem that affects survival. For example, the 5-year survival rate for patients diagnosed with metastatic cervical cancer is 16.5% compared to 91.5% for localised cervical cancer. The treatment used in the early stages includes surgery and radiation therapy, and for advanced stages, radiation therapy is combined with cisplatin-based chemotherapy. However, these treatments can only be effective in the early stages and when the patient has limited metastasis. Furthermore, essential data to consider are the relapse rates in patients with cervical cancer, which are low for patients with limited disease and high for patients with metastatic stages (recurrence rate per stage: stage IB-around 10%, for stage IIA-17%, stage IIB-23%, stage III-42% and stage IVA-74%). Thus, the high relapse and mortality rate is associated with the development of immune system evasion, drug resistance, invasion, and metastasis. From these data arises the need for targeted immunotherapies, which have increased in recent years. However, the therapeutic options for cervical cancer in advanced stages continue to be limited [15,16,17,18,19].

## 2. HPV

Human papillomavirus (HPV) infection is linked to several cancers, including cancers of the cervix, vagina, vulva, head and neck, anus, and carcinomas of the penis. HPV belongs to the Papillomaviridae family-circular double stranded deoxyribonucleic acid (DNA) viruses, without an envelope of approximately 8000 base pairs. There are more than 200 types of HPV, and according to their sequence, they can be divided into alpha, beta, gamma, delta, and mu. Papillomaviruses that infect the uterine cervix belong mainly to the alphavirus genus. HPV DNA contains early and late genes that code for six early proteins (E1, E2, E4, E5, E6, and E7) and two late (L1 and L2). The E1 and E2 proteins are necessary for the replication and translation of the virus. E2 also regulates the expression of E6 and E7; E4 and E5 help in viral assembly and growth stimulation, while the late L1 and L2 proteins form minor and major capsid proteins. HPV proteins can also be divided into core and accessory proteins. The core proteins (E1, E2, L1, L2) are directly involved in the replication of the viral genome (E1, E2) and the assembly of the virus (L1, L2), generally reflecting when transcription occurs during the viral life cycle. On the contrary, accessory proteins (E4, E5, E6, and E7) show more significant variability in their moment of expression and functional characteristics. The genes that encode accessory proteins modify the infected cell to facilitate viral replication in ways that correlate with each other’s different disease associations [1,19,20].

### 2.1. HPV Infection and Transformation

The transformation capacity of HPV results mainly from the activity of the E6 and E7 oncoproteins, which impair growth regulatory pathways. The persistence of high-risk HPV can progress from a productive infection (producing virions) to an abortive or transforming infection (when genetic material is integrated into the host cell’s DNA), then cancer may develop after an accumulation of host genetic changes, including chromosome abnormalities, point mutations and changes in patterns of host gene methylation [21,22].

For the development of productive infection, HPV must infect basal layer keratinocytes, which can proliferate. Once HPV is established in basal cells, HPV activates an early promoter that induces the expression of viral genes that aid in the replication and establishment of the virus. The first proteins that are expressed are E1 and E2, which are necessary to initiate replication. In this way, the viral episomes are amplified in undifferentiated cells, where viral genomes are maintained due to the replication of cellular DNA. The infected basal cells divide, and the viral DNA divides into daughter cells. Some cells migrate from the basal layer initiating differentiation, inducing the productive phase of the viral life cycle by activating the late promoter and the expression of the viral genes E4, E5, L1, and L2, which also increases the expression of E1 and E2 and the amplification of the viral genome with the subsequent assembly and release of virions in the upper layer of the epithelium [21,22,23,24]. In HPV-infected cells, the E6 and E7 proteins deregulate the normal cell cycle checkpoints, inducing uncontrolled proliferation and preventing the activation of apoptosis, since E6 and E7 target the tumour suppressor proteins p53 and pRb, respectively. E7 promotes the degradation of pRb, induced by the release and activation of the E2F transcription factor promoting the re-entry into the S phase of differentiated cells to promote their replication. Usually, unscheduled re-entry of the cell cycle would activate p53 to induce cell cycle arrest, damage repair and, if this is not possible, apoptosis in infected cells. However, E6 avoids these events by targeting p53 for degradation [25]. In the viral cycle, the E2 protein (transcriptional repressor of E6 and E7) controls the expression of E6 and E7. However, in carcinogenesis, the HPV genome is often integrated into cellular genetic material (abortive infection). Integration disrupts the E2 gene, resulting in increased expression of the E6 and E7 oncoproteins and cell transformation. Much of the sexually active population will experience transient RA-HPV infections during their lifetime. Still, these infections will not progress to a clinically significant lesion or cervical cancer since the immune system can recognise the virus and virus-infected cells and remove them. Cancer progression is due to persistent infection with RA-HPV and evasion of the immune system [25,26,27,28].

### 2.2. Activation of the Immune System in HPV Infection

The activating response of immune cells to an HPV infection starts with keratinocytes, which are part of the innate immune system and play a role as non-professional antigen-presenting cells. Keratinocytes can present viral peptides in class 1 histocompatibility molecules, since HPV multiplies within these cells without inducing lysis. However, although keratinocytes do not express HLA class II molecules at baseline, they can do so when stimulated with cytokines such as interferon gamma (IFN-γ). It seems that this capacity to express MHC class II plays an essential role in the accumulation and control of Th1 cells. On the other hand, keratinocytes can express different pattern recognition receptors such as Toll-like receptors (TLRs); for example, TLR9 can recognise double-stranded DNA. In response to this recognition, keratinocytes can produce cytokines such as interferon (IFN) of type 1, tumour necrosis factor alfa (TNF-α), interleukin-18 (IL-18), and specific chemokines such as CCL2, CCL20, CXCL9. These cytokines and chemokines facilitate the recruitment and activation of more immune system cells such as NK, NKT, LCT CD4+ and CD8+ cells, macrophages, dendritic cells, among other cells important in activating the immune system [29,30,31].

In contrast, the mucous epithelial layers have resident and transient professional antigen-presenting cells (APC), which are also part of the innate immune system and are necessary for activating the cells of the adaptive immune system for a response to be achieved by T cells and subsequent B-cell activation and antibody production. APCs comprise macrophages and dendritic cells (DCs), which capture and present antigens, in this case, HPV or cells infected with HPV to later present the antigens to T cells. APCs express pattern recognition receptors (PRRs) capable of recognising HPV and, after recognising pathogens and their activation, can express co-stimulatory molecules and cytokines necessary for activating CD4 and CD8 T cells [9,12,32,33].

**Figure 1:**
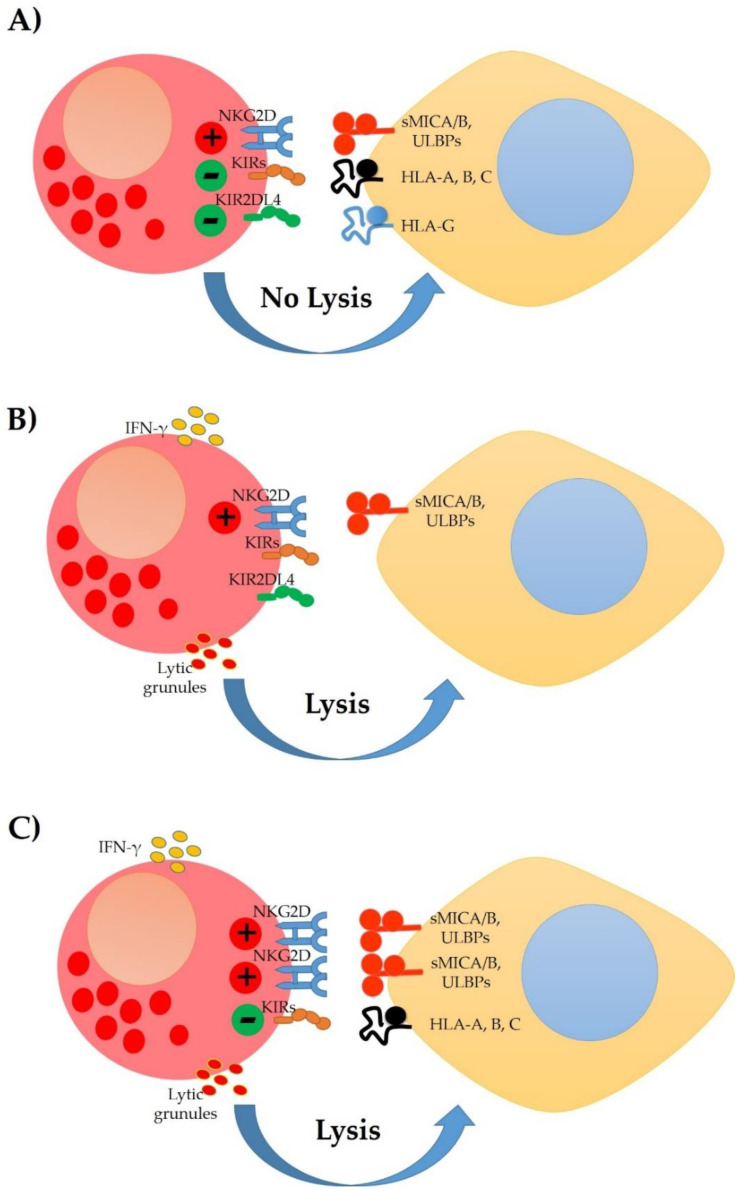
Natural killer (NK) cells express activator and inhibitory receptors; their activity depends on balancing these interactions with their respective ligands. (**A**) When signalling via inhibitory receptors exceeds signalling via activating receptors, the activation of NK cells is inhibited, and tolerance is generated. (**B**) When target cells decrease the expression of inhibitory ligands (HLA-A, B, C) and increase the expression of stimulatory molecules (MICA/B, ULBPs) and these interact with the activating receptors of NK cells such as NKG2D, the result is receptor activation that release cytokines from NK cells and cytotoxicity against the target cell. (**C**) When the target cells express a greater amount of stimulator molecules (MICA/B, ULBPs), the active signalling exceeds inhibitory signalling, leading to NK cells’ activation.

There are different ways in which NK cells can be activated and eliminate target cells. Such activity is regulated by the presence of inhibitory and activating receptors on the cell surface (Figure 1). For example, MHC class I molecules, which generally are present in all nucleated cells, bind to inhibitory receptors (such as KIR-L and NKG2A) to inactivate NK cells. When MHC molecules are not abundant (a common event in HPV-infected cells and tumour cells to evade the LcT response), the NK cells become activated due to the absence of inhibitory signals. Another way of activating NK cells is by recognising damage or stress receptors. Cervical cancer cells express these types of receptors. For example, they express MICA/MICB and ULBPs (1–6), molecules recognised by the NKG2D receptor. CD95 recognises CD95L, B7-H6 interacts with NKp30, the Fc fractions of IgG antibodies are recognized by CD16 to activate the cytotoxicity of NK cells, as well as CD112 and CD155 (usually expressed in tumour cells), which are recognized by DNAM-1 (Table 1), whose interaction also promotes cytotoxicity and the generation of cytokines. In addition, NK cells express activation markers such as NKp46 and NKp44 capable of interacting with viral hemagglutinin and neuraminidase, encoded by foreign pathogens. Receptor-ligand interactions activate NK cells with the subsequent elimination of the target cell. On the other hand, NK cells produce large amounts of interferon-gamma that plays a relevant role in activating the innate immune system and differentiating T helper cells [14,34,35,36,37].

NK cell receptors can promote cell inhibition or activation, and these events depend on the cytoplasmic domains present on these receptors and the kinases with which they are associated. For example, some inhibitory receptors (NKG2A and NKG2B) have motifs in their intracytoplasmic domains called ITIM (inhibitory immunoreceptor motifs based on tyrosine). These motifs can bind to the SH2 domain associated with tyrosine phosphatases and, thus, promote the inhibition of cellular cytotoxicity by dephosphorylation. On the contrary, NK cells also have activating receptors (NKG2D), which lack ITAM motifs (tyrosine-based immunoreceptor activation motifs) but can associate with the DAP-12 molecule, which has ITAM sequences to which tyrosine kinases bind, such as kinases of the Syk family, and thereby promotes the activation of NK cells [38,39,40].

## 3. NK Cells Populations

**Figure 2:**
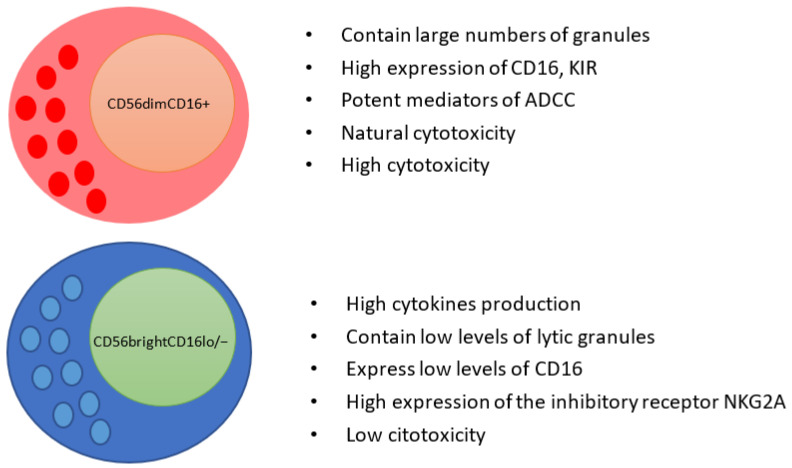
CD56bright and CD56dim NK cell subpopulations express differential receptor profiles and have different innate immune functions. CD56brightCD16low/− NK cells secrete large amounts of cytokines but low expression levels of lytic granules and the FcγRIII (CD16). In addition, they have a high expression of the inhibitory receptor NKG2A. In contrast, CD56dimCD16+ NK cells contain large numbers of granules, express high levels of CD16, KIR, are potent mediators of ADCC, LAK activity, and natural cytotoxicity.

Natural killer (NK) cells represent approximately 10% of peripheral blood lymphocytes. These cells are highly relevant innate lymphocytes, a central function is cytotoxicity without pre-sensitisation, and they produce large amounts of inflammatory cytokines, such as IFN-γ and TNF-α. NK cells are generally identified by flow cytometry, using three markers. The first requirement is the lack of expression of the T lymphocyte marker (CD3), and the second is the expression of CD56 (neural cell adhesion molecule 1, NCAM1), and CD16 (low-affinity Fc gamma receptor 3A, FcγRIII). Human NK cells have generally been divided into two main subpopulations based on differential expression of CD56 and CD16: (1) CD56bright/CD16low and (2) CD56dim/CD16+ (also classified as CD56bright and CD56dim, respectively) (Figure 2). Cytotoxicity and the ability to produce cytokines in these two subpopulations is differential. For example, CD56dim/CD16+ cells have a greater cytotoxic capacity and express higher Ig-like NK receptors (NKp44, NKp30, and NKp46) and FcγRIII (CD16) that facilitate their antibody-dependent cytotoxicity. In contrast, CD56bright/CD16low NK cells can produce more cytokines, but their cytotoxic activity is low [34,41,42].

## 4. Modulation of the NK Cells Response in Cervical Cancer

NK cells play an essential role in recognising and eliminating cervical tumour cells and, thus, cells infected by the HPV virus. However, cervical tumour cells have developed mechanisms to evade NK cells attack, and HPV and its oncoproteins contribute to the evasion process of the immune system to prevent the elimination of tumour cells. We will describe the strategies used by cervical tumour cells and the contribution of HPV in the evasion of the immune system by NK cells.

NK cells play a role in the surveillance against HPV infection, the development and progression of cervical intraepithelial neoplasms (CIN), and cancer of the uterine cervix. Typically, NK cells infiltrate the uterine cervix, and they are present when cervical lesions develop. Nevertheless, their activity can be achieved according to the cervical cancer stage. This phenomenon was reported by the group of Vaquer et al. 1990 who analysed the cytotoxic activity of peripheral blood NK cells of patients with cervical cancer in different stages; they observed that peripheral blood NK cells from patients with localised uterine cervix carcinoma stages (I, II, III and IVa) had a cytotoxic activity very similar to that of healthy women. However, cytotoxic activity decreased in patients with metastatic stages (IVb). It is important to emphasise that this study was performed with peripheral blood NK cells and that the presence or type of HPV was not considered.

Alternately, studies have reported infiltrating NK cells in the cervix with the CD56brightCD16- phenotype, which could respond to the positive regulation of the CD155 ligand DNAM-1 and the MICA ligand of NKG2D that have been reported in cervical cancer biopsies. However, the presence of HPV could modulate the amount and cytotoxicity of NK cells present in cervical tissue, as shown by a study that reported a higher percentage of NK cells in low-grade cervical lesions with a low viral load. In addition, some reports show that HPV16+-infected women have a higher amount of NK cells compared to cells obtained from cervical brushing of HPV18+-positive patients. However, these cells do not express IL-2 and have a low cytotoxic capacity. These data correlated with the observation that there are more severe lesions of the cervix in women with HPV16+ compared to women with HPV18+. This observation indicates the contribution of HPV to the evasion of the NK cells response [43,44,45,46,47].

On the other hand, it has also been observed that tumour cells can secrete viral oncoproteins E6 and E7 and that these soluble proteins affect NK cells. They reduce IFN-γ induced by IL-18 because E6 and E7 bind to the IL-18 receptor. This union prevents the cytokine from binding to its receptor and activating the IRAK/TRAF6 pathway resulting in the activation of NF-κB and AP-1, which promote IFN-γ transcription. IFN-γ is an essential cytokine that activates NK cells and other immune system cells such as CD8, NKT and Th1 cells. Inhibiting the production of IFN-γ activates a wide range of cells. Moreover, IL-18 signalling plays a vital role in the activation of NK cells since it promotes their expansion and improves their cytotoxicity and tumour activity, and the expression of CD80, CD86, HLA-DR and HLA-DQ [48,49].

Another way viral oncoproteins can affect the production of cytokines necessary for the activation of NK cells is by avoiding the activation of the inflammasome, which is necessary for the production of IL-18 and IL-1β. Song et al. showed that the E7 oncoprotein is capable of interacting with IFI16 and TRIM21 and that the HPV E7 protein was also able to recruit the E3 ligase TRIM21 to ubiquitinate and degrade the IFI16 inflammasome. These data indicate that viral oncoproteins not only affect IFN-γ production by reducing IL-18 signalling but are also capable of degrading the inflammasome to decrease IL-18 directly. IL-1β also co-stimulates IFN-γ production by NK cells. Viral oncoproteins (E6VPH16) reduce the amount of IL-1β by inhibiting the transcription of IRF6 by degrading p53 and thus decreasing the transcription of IL-1β [50,51,52].

One strategy of cervical tumour cells to evade NK cells response is to inhibit the production of cytokines necessary for their activation and proliferation. Moreover, a decrease in the activation of molecules such as NKp30, NKp46 and NKG2D on NK cells surface decrease their cytotoxic capacity. Tumour cells can secrete regulatory cytokines such as transforming growth factor beta (TGF-β) and interleukin-10 (IL-10) that reduce the activation of NK cells. For example, TGF-β binds to its receptor and activates the phosphorylation of SMAD2/3, essential in regulating gene expression; IL-10 interaction with its receptor activates STAT3, SMAD-2,3,4, and STAT3 are transcription promoting factors that are associated with anti-inflammatory and tolerogenic responses. Other strategies of tumour cells are the overexpression of non-classical HLA such as HLA-G that can interact with the KIR2DL4 receptor (inhibitory receptor with ITIM motifs) and prevent the NK cells activation.

**Figure 3:**
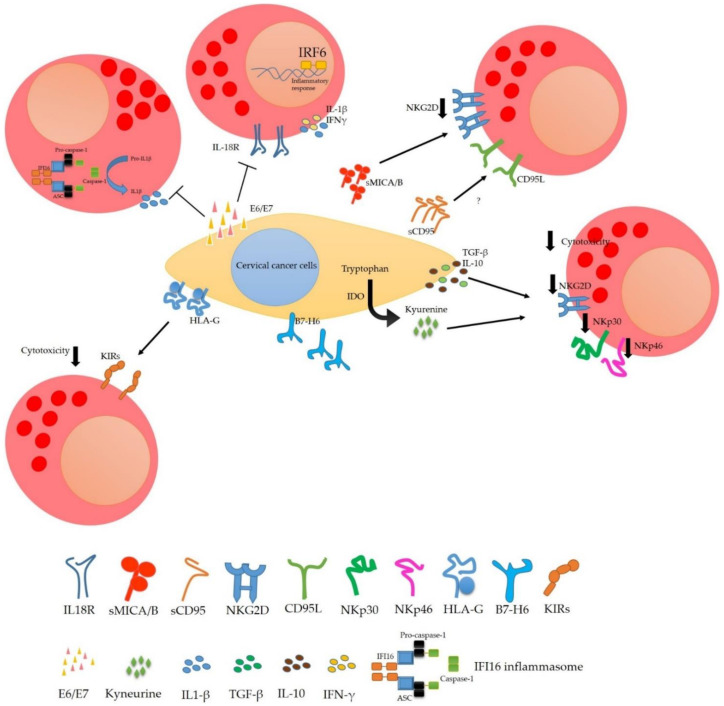
Evasion the immune response of NK cells in cervical cancer. Cervical tumour cells have developed mechanisms to evade NK cells’ attack. The viral soluble oncoproteins E6 and E7 affect NK cells because E6 and E7 bind to the IL-18 receptor and thus prevent IL-18 from binding to its receptor with subsequent signalling reducing the amount of IFN-γ. E7 oncoprotein can induce the degradation of the IFI16 inflammasome: this affects IFN and IL-1β production. The tumour cells can secrete regulatory cytokines such as TGF-β and IL-10 and release damage or death ligands like MICA/B and CD95 that reduce the activation of NK cells. Moreover, cervical cancer cells express some enzymes such as IDO, which degrades tryptophan and leads to the production of immunosuppressive kynurenines, which can inhibit the activation of NK cells. Moreover, inducing a decrease in activating receptors NKG2D and NKp46 expression affects the cytotoxicity of NK cells and their ability to produce inflammatory cytokines.

On the other hand, the release of damage or death ligands (MICA/B, CD95) can function as decoys and lysis of the tumour cell is avoided. Other mechanisms are poorly understood, but we believe they participate in the evasion of the immune response. For example, our group demonstrated that cervical tumour cells express NK cells markers such as NKG2D, NKG2A, NKp30, NKp46 mainly. However, we do not know why cervical cancer cells express these molecules and the benefit they bring to tumour cells regarding NK cells activity (Figure 3) [35,53,54,55,56,57,58,59,60,61,62].

Enzyme expression also plays an essential role in inhibiting the activation of the immune system. Cervical tumour cells can express the immunomodulatory enzyme indolamine-2,3-dioxygenase (IDO), which degrades tryptophan and produces immunosuppressive kynurenines. Furthermore, it has also been observed that the viral oncoproteins E6/E7 may regulate the expression of this enzyme. In patients with cervical cancer, IDO expression has been correlated with decreased disease-free survival and overall survival. The activity of IDO generates L-kynurenine as a secondary catabolite, which can inhibit the proliferation of NK cells, inducing a decrease in the expression of the activation receptors NKG2D and NKp46, affecting the cytotoxicity of NK cells and their ability to produce inflammatory cytokines such as IFN-γ and TNF-α [63,64,65,66,67,68].

## 5. NK Cells and Immunotherapy in Cervical Cancer

Immunotherapy represents a novel approach to treat cancers based on the immune system’s natural ability to recognise and eliminate tumour cells. In particular, there is significant interest in immunotherapy for cervical cancer due to the viral antigens that could be recognised as foreign and thus represent an attractive treatment option. While checkpoint inhibition by PD-1 receptor blocking is the main class of drugs tested in cervical cancer, several other immunotherapy strategies are under development. However, limited studies or clinical trials are underway for NK cells as an immunotherapy option.

NK cells can eliminate tumour cells that express surface markers associated with oncogenic transformation, express death ligands, or decreased expression of MHC molecules. In addition, their antibody-dependent cytotoxicity and ability to produce inflammatory cytokines—that cooperate with the activation of other cytotoxic cells of the immune system—provide NK cells with the ability to serve as anticancer agents. However, as previously discussed, tumours can develop several mechanisms to evade the response of NK cells; for this reason, different strategies to promote the antitumor response of NK cells have developed recently. Such therapies are based on inhibitors, antibodies, and other drugs that seek to improve the activation of NK cells and tumour elimination. Other therapies are explored based on the expansion and activation of NK cells and ex vivo genetic modification of autologous or allogeneic NK cells. These strategies are currently being evaluated in clinical trials, including patients with solid tumours and haematological neoplasms [69,70,71,72,73,74]. In some cases, the results show remissions either complete or partial. These encouraging data provide a rationale for the analysis of NK cells as strategic components in the therapy against different types of cancer continues [75].

### 5.1. Treatments That Enhance NK Cell Activity

NK cells play a crucial role in eliminating cervical tumour cells, and nowadays there are many publications on how tumour cells can be treated to increase their recognition by NK cells. In contrast, there are also studies on the use of autologous or allogeneic NK cells and their various treatments to increase their activation or response against NK cells. This section will discuss these types of strategies used to enhance NK cell activity and immunotherapy.

For example, the in vitro treatment of cervical tumour cells with vorinostat (a histone deacetylase inhibitors) has been shown to promote an increase in MICA expression that enhances the NK cell-mediated cytolytic reaction [76]. In other neoplasms such as myelodysplastic syndrome, the use of vorinostat is followed by the administration of NK cells (NCT01593670) is being studied. However, in cervical cancer, only one clinical study is evaluating its administration combined with pembrolizumab that is in progress (NCT04357873), and that does not consider the administration or analysis of the activity of NK cells.

The inhibition of IDO could be a useful strategy by which to enhance the response of NK cells to cervical tumour cells. The group of Sato et al. demonstrated that cervical cancer cells express the enzyme IDO and that its inhibition with interfering RNA improves the response of NK cells in vitro and in vivo, promoting the better elimination of tumour cells and a reduction in tumour size associated with NK cell infiltration. Moreover, a phase one study (NCT03192943) using the IDO inhibitor (BMS-986205) combined with nivolumab (anti-PD-1) in patients with advanced tumours has also been investigated. According to data from Jason Luke, the combination of these treatments in 22 patients with cervical cancer showed an objective response rate of 14% and a durable response rate of 64%. It also showed that the combination is not toxic and that it can be a new immunotherapy option. Jason Luke mentions that this treatment reduced the serum levels of kynurenine and induced an increase in the number of cytotoxic T cells in most of the patients. Analysis of the effect of this immunotherapy on NK cells has yet to be conducted [77,78,79]. Another enzyme involved in the evasion of the response of NK cells, and that is expressed in cervical tumour cells, is haem oxygenase 1 (HO-1). Studies by Gómez-Lomelí et al. showed the importance of HO-1, an enzyme associated with the regulation of NK cells, and how its inhibition improves the cytotoxic response of NK cells against cervical tumour cells. They demonstrated the expression of the HO-1 in SiHa, HeLa and C33A cell lines and how its expression increases in HPV+ cell lines. They also showed that when tumour cell lines are pre-treated with HO-1 inhibitors and co-cultured with NK92 cells, the percentage of NK92 CD107 cells positive for IFN-γ and TNF-α increases. In contrast, they also showed the downmodulation of NCR (Nkp30 and NKp46) and NKG2D in NK-92 cells and NK cells of healthy donors treated with the supernatant of HeLa, SiHa, and C-33A cervical cancer cells and how the pre-treatment with the HO-1 inhibitor restored the expression of NKG2D and NKp30. These data could point to HO-1 inhibition as a therapeutic target. However, it is necessary to analyse whether pre-treatment of cervical tumour cells with HO-1 inhibitors improves the cytotoxicity of NK cells or if the better production of IFN-γ and TNF-α impacts the activation of other cells of the immune system [80,81].

Another mechanism used by cervical tumour cells is the expression of matrix metalloproteinases and disintegrins, such as MMP14, ADAM9, ADAM10 and ADAM17. These proteases can cleave the ligands of NKG2D (MICA/B) and NKp30 (B7-H6) from the cell surface to prevent recognition by the NK cells. The ADAM9 inhibition with miR-126 suppresses the proliferation of tumour cells and improves the sensitivity of these cells against chemotherapeutic drugs; in addition, some studies reported that the overexpression of ADAM17 is associated with aggressive cancers and a bad prognosis. Therefore, the inhibition of these proteases could represent a novel approach in treating patients with cervical cancer. However, there are limited studies on the relevance of these proteases in tumour cell biology, and there is no evidence of whether the use of ADAM inhibitors improves the cytotoxicity of NK cells versus tumour cells. Remarkably, there are reports on the role of treating NK cells with different ADAM inhibitors to avoid the cleavage of cell surface receptors such as CD16 (FcγRIII), CD62L, among others. One of these antecedents was carried out by Mishra et al., who showed that treating NK cells with MEDI3622 (anti ADAM17) in combination with IL-15 increases the proliferation of NK cells in vivo and in vitro models. On the other hand, Pham et al. showed that the treatment of NK cells with the ADAM17 inhibitor (TAPI-1) increases the purity of NK cells expanded ex vivo, the expression of CD16 (FcγRIII), IFN-γ production and improves antibody-dependent cytotoxicity activity against breast cancer cells. These data do not indicate that blocking ADAMs proteases may have a dual role and provide a new approach enhancing the persistence and function of NK cells in cancer patients [82,83,84,85,86,87,88,89,90].

Another molecule important in the development of cervical cancer and the immunomodulation of the tumour microenvironment is STAT3, a protein that is over-expressed in HPV+ cervical cancer cells; its inhibition induces a decrease in the proliferation of these tumour cells and even reverses resistance to cisplatin. In the context of NK cells, inhibition of STAT3 enhances the expression of granzyme B, perforin, and DNAM-1 and improves their in vitro cytotoxicity against leukaemia, lymphoma and melanoma cells. However, there are no studies on the effect of STAT3 inhibition in NK cells and its response against cervical cancer cells [61,91,92].

In conventional medicine, different techniques seek to promote the increase in the number and activity of NK cells in various types of cancer. However, alternative medicine methods such as electroacupuncture (EA) have been studied, along with their impact on the activation of NK cells. In this context, Saraswati et al. showed that patients with cervical squamous cell carcinoma (SCC) (stages IIb–IIIb) who had chemotherapy and EA had significantly increased NK-cell percentages in their peripheral blood and a significant decrease in their cervical tumours compared to the control group (who only received the chemotherapy). These data correlate with those of Zhang et al., who observed that the EA relieved tumour growth in breast tumour-bearing mice by alleviating inflammation and enhancing antitumor immunity, mediated by promoting CD8+ T cells and NK cells. They showed how EA increases the number of NK cells in peripheral blood, infiltrates the tumour, and increases granzyme B and perforin in the tumour tissue. These findings suggest that EA could be a complementary alternative in cancer treatment, however, more studies are required [93,94,95].

### 5.2. Therapies Based on the Infusion of NK Cells in Cervical Cancer

Therapies based on the transfer of NK cells are another alternative analysed in treating various haematological cancers and solid tumours. These are based on the use of autologous, allogeneic, haploidentical NK cells or derived from umbilical cord progenitor cells which are treated with IL-2, IL-15, IL-18 or stimulatory cells (K562 expressing mbIL -21, mbIL-15), and after the infusion process, the patient is treated with recombinant IL-2 or IL-15. The objective of these therapies is to transfer to the patient NK cells capable of recognising tumour cells and eliminating them. With these strategies, it has been observed that some patients achieve total remission, partial remission, or stable disease. These results have encouraged the investigation of the transfer of NK cells in other types of cancers [16,75,96]. In this context, there are limited studies to analyse the effect of NK cells in cervical cancer cell infusion and the immune response. Veluchamy’s group, in 2016, evaluated in vitro the effectiveness of using allogeneic NK cells derived from CD34+ progenitor cells from umbilical cord blood (UCB-NK) and peripheral blood NK cells (PBNK) with or without cetuximab (anti-EGFR) against different cervical cancer cell lines. Their results showed more significant cytotoxic activity in UCB-NK cells compared to PBNK. The activity of UCB-NK was independent of the expression level of HLA-A, -B or -C. These data suggest the importance of analysing NK cell immunotherapy to treat patients with cervical cancer [97]. However, there are only two ongoing investigations available in https://clinicaltrials.gov/ (accessed on 1 November 2021). One (active, not recruiting) raises the analysis of radiofrequency ablation and the transfusion of cytokine-induced killer cells (NCT02490748); the other (complete but without results) raises the analysis of cryosurgery plus NK cell immunotherapy for recurring cervical cancer (NCT02849340). It is essential to mention that cryosurgery combined with allogeneic NK cell immunotherapy in the treatment of advanced kidney cancer showed promising clinical efficacy and safe treatment [98]. The information reviewed in this article highlights the need for focusing research on using NK cells to treat cervical cancers.

## 6. Novel Cellular Immunotherapies to Treat Cervical Cancer

Human NK cells are cells of the innate immune system that act as effector cells and play an essential role in the immune response against tumours. NK cells can directly exert their cytotoxic function by releasing perforins and granzymes by activating their cytotoxicity dependent on antibodies to eliminate tumour cells. Indirectly, NK cells can generate cytokines such as IFN-γ, necessary for the activation of other cells of the innate and adaptive immune system. Tumour cells create a tumour microenvironment that allows them to escape the cells’ recognition of the immune response, generating immune tolerance. However, tumours can interrupt the immune tolerance, and in this way, can promote the activation of cells of the immune system and promote the elimination of tumour cells. In this context, there is currently great interest in analysing chimeric antigen receptor (CAR)-engineered NK cells for application in tumour immunotherapy. The use of chimeric receptors on NK cells allows targeting specific antigens, such as tumour-specific antigens or tumour-associated antigens. The objective of modifying NK cells with chimeric antigen receptors (CAR-NK) is to facilitate the recognition of tumour cells. This modification can influence improved proliferation, persistence and increase in NK-CAR cells infiltrated in the tumour; these events disrupt the tolerogenic tumour microenvironment, thereby achieving a better activation of the immune system and an adequate elimination of tumour cells. However, there are some difficulties in using CAR-NK cells; one is the identification of exclusive tumour antigens or tumour-associated antigens against which CARs can be designed so that these cells do not react against their antigens. Another critical aspect to consider is the source of NK cells. Some NK cell sources are cell lines, peripheral blood NK cells, autologous or allogeneic NK cells derived from the umbilical cord, bone marrow progenitors or cells of mobilised peripheral blood. It is important to consider that each of these sources has different limitations, such as their low expansion potential, immaturity, absence of maturation receptors, or low cytotoxicity. That is why many in vitro and in vivo studies analyse the use of CAR-NK to treat different tumours such as breast, ovarian, lung, liver, gastric cancer, glioblastoma, among others. The results of these studies to date are encouraging due to the observed increase in cytotoxicity, decrease in tumour mass, increase in IFN-γ, granzymes and perforins, and increase in survival. However, in cervical cancer, we only have the antecedent of a study carried out by Huan et al., where they used the NK92 cell line and modified them with a CAR directed against the prostate stem cell antigen (PSCA). This group mentioned that the targeting of CAR towards PSCA was performed because they evidenced the presence of this antigen in the cervical cancer lines HPV18+ HeLa and MS751 in 100 and 83.6%, respectively. Later, they analysed the effect of the PSCA CAR-NK92 cells in vitro and in vivo. In vitro, they found that PSCA CAR-NK-92 cells were capable of lysing HeLa and MS751 cells in a dose-dependent manner but were not capable of lysing cells that did not express PSCA. Furthermore, the cultivation of PSCA CAR-NK-92 cells with MS751 cells induced an increase in the secretion of IL-2, IFN-γ and TNF-α. Finally, in the in vivo model, they generated tumours in nude mice with the MS751 cell line and observed a significant decrease in tumour mass compared to the control (NK-92), which correlates with PSCA CAR-NK-92 cells. This study implies the need for the analysis of CAR-NK cells in the treatment of cervical cancer [99,100].

Gene therapies are another alternative to achieve the expression of activator molecules in NK cells and thereby improve their cytotoxicity. Under this context, Huang et al. analysed the preassembled CRISPR-Cas9 ribonucleoprotein nucleofection (Cas9 RNP) to insert promoters to reactivate silenced genes in NK92 cells, known to be less cytotoxic cells than primary NK cells, due to the silencing of some genes. The insertion of promoters was carried out by designing a homology-directed repair (HDR) mediated by Cas9 to reactivate endogenous genes by replacing the silenced promoter with a promoter from the spleen focus-forming virus (SFFV). In this way, they reactivated the expression of DNAM-1 in NK92 cells, after which NK92 DNAM-1+ cells were challenged against HeLa cervical cancer cells and had four times higher cytotoxicity than NK92 cells. These data highlight another promising strategy that should be considered for analysis in vitro and in vivo experimental models [101].

Analysing the use of NK cells as a tool for targeted therapy is an excellent strategy since these are cells of the adaptive immune response with a high immediate lytic capacity. However, tumour cells moderate the tumour microenvironment and the expression of their receptors to avoid recognition by cells and components of the immune system, making cells tolerogenic, anergic, or even inducing apoptosis. Thus, it is necessary to reverse this lack of response in NK cells to recognise tumour cells and achieve their elimination. Today, there is extensive research on many types of cancer that use NK cells from human cell lines (NK92), peripheral blood or derived from progenitors of bone marrow, umbilical cord or mobilised peripheral blood and that also consider the treatment of NK cells ex vivo with growth factors and cytokines for promoting their activation. Another alternative is gene therapy, inducing the expression of specific receptors to recognise tumour-associated antigens or through the insertion of promoters that promote the overexpression of activating receptors; these strategies have shown encouraging results. However, some points must be considered, such as the most optimal form of administration, dose, periodicity, and whether they need administration of exogenous cytokines for their maintenance. Other questions are whether NK cells will infiltrate the tumour, whether their activated phenotype is maintained in the tumour microenvironment, and whether they can generate unwanted reactions to recognise normal cells. Unfortunately, the investigation of these alternatives in cervical cancer is understudied. What is known so far is that treatment with specific inhibitors such as vorinostat, pembrolizumab, IDO inhibitor, HO-1 inhibitor improves the cytotoxicity of NK cells in cervical cancer [76,79,81,98].

On the other hand, few studies have focused on using NK cells as a potential therapy in the treatment of cervical cancer. The reported studies propose using allogeneic NK cells derived from CD34+ progenitor cells from umbilical cord blood (UCB-NK) or obtained from peripheral blood (PBNK). Another study suggests using the genetically modified NK92 cell line to express a CAR (PSCA CAR-NK-92) and another genetic modification to promote activator receptors (NK92 DNAM-1+). These strategies have shown encouraging results since they show improved cytotoxicity against cervical tumour cells. The study of targeted therapy using NK cells is a highly productive field in all cancer types, especially cervical cancer. Particular attention should be focused on STAT3 and its effect on the activation of NK cells and cytotoxicity against cervical tumour cells, as well as the search for more tumour-associated antigens for the generation of CAR-NKs and the implementation of Cas9 RNP to reactivate silenced relevant genes in the cytotoxicity of NK cells against cervical tumour cells [98,100,101].

## 7. Conclusions

NK cells are a critical heterogeneous subpopulation of the innate immune response. They are capable of directly recognising and eliminating tumour cells without the need for pre-sensitisation. NK cells can also produce cytokines that enhance the activation of other immune system cells, either innate or adaptive cells. Recently, great emphasis has been placed on the study of NK cells and their use in immunotherapy for the treatment of different cancers, among which the most studied are haematological cancers. Encouraging results have been found; however, a greater focus on each application of these cells—autologous, haploidentical, allogenic or derived from hematopoietic progenitors stimulated ex vivo for their subsequent infusion in patients with neoplasms—is required. Research on cervical cancer, a highly prevalent cancer worldwide, has shown that these tumour cells generate various strategies to decrease the activation and cytotoxicity of NK cells. Various in vitro and in vivo treatments have shown that the activation and cytotoxicity of NK cells can be increased to promote tumour elimination. However, research regarding the use of NK cells in immunotherapy is limited. Clinical studies that can uncover the safety and feasibility of their use to promote a better response in patients with cervical cancer are needed.

## Figures and Tables

**Table 1:** Ligands of human NK cell receptors.

Receptor		Ligand
**Activating Receptors**		
	NKp30	B7-H6, BAG6, Galetin-3, heparan sulfate proteoglycan (HSPG)
	NKp44	Viral hemagglutinin (HA), haemagglutinin-neuraminidase (HN), glycoproteins and proteoglycans, nuclear proteins that can be exposed outside the cell
	NKp46	HA, HN, heparan sulfate (HS), glucosaminoglycans (GAGs)
	NKp80	activation-induced C-type lectin (AICL)
	KIR-S	HLA-C, HLA-B
	NKG2C	HLA-E
	NKG2D	MICA/B, UBLP1-6
	NKG2E	HLA-E
	CD2	CD48
	CD16	Fc IgG
	CD95L	CD95
	CD96	CD155
	CD226 (DNAM-1)	CD112, CD155
**Inhibiting Receptors**		
	KIR-L	HLA-A, B, C
	NKG2A	HLA-E
	NKG2B	HLA-E
	TIGIT	Nectin 4, CD112, CD155
	PD-1	PDL1

## Data Availability

Not applicable.
